# Supplementary material for: Complexome profiling of the Chlamydomonas psb28 mutant reveals TEF5 as an early PSII assembly factor
Source: Plant Cell. 2025 Mar 17;37(6):koaf055. doi: 10.1093/plcell/koaf055 (PMC12164590; doi:10.1093/plcell/koaf055)
Supplement: koaf055_Supplementary_Data [file koaf055_supplementary_data.zip › psb28_interactive.zip!/plots/intensity/qualityPlots/peptides/FAP358_Cre12.g493250.t1.2.html]

### Cre12.g493250.t1.2

Trivial name: FAP358  
SeqCoverage [median]: 26.10  
Mass: 81.03 kDa  
  
 

| Sequence | MissedCleavage | Mass [Da] | wtIntensity | muIntensity | Score |
| --- | --- | --- | --- | --- | --- |
| ADATDYEFIK | 0 | 1171.54 | 712.92 | 2808.12 | 105.10 |
| DLKPENVLIDGQGYVK | 1 | 1786.95 | 4149.55 | 0.00 | 58.08 |
| EGEAQVIQGDREVNR | 1 | 1698.83 | 3788.33 | 7264.51 | 83.73 |
| EKSPEVVNQR | 1 | 1184.61 | 497.70 | 529.25 | 85.36 |
| IGMLQGK | 0 | 745.42 | 0.00 | 622.10 | 98.51 |
| IVAEMYALPVQAGEILIQQGDSGDAATK | 0 | 2887.46 | 664.16 | 0.00 | 77.51 |
| KLVLVEGELLGEGAFSR | 1 | 1816.01 | 579.37 | 0.00 | 45.62 |
| KTELAEAHR | 1 | 1053.56 | 331.58 | 470.86 | 99.34 |
| SGKFEVLER | 1 | 1063.57 | 678.44 | 2396.86 | 82.29 |
| SPEVVNQR | 0 | 927.48 | 530.20 | 0.00 | 89.14 |
| SSLVNLLLFSR | 0 | 1247.72 | 2407.86 | 0.00 | 126.19 |
| TQDPMEVMRR | 1 | 1261.59 | 0.00 | 493.71 | 42.40 |
| VIMEGDVGDKFYIIK | 1 | 1725.90 | 4073.07 | 2298.92 | 61.38 |
| VTEETTGR | 0 | 891.43 | 0.00 | 531.38 | 71.38 |
|  |  |  | 18413.2 | 17415.7 |  |
